# Some Like It Hot: Maternal-Switching With Climate Change Modifies Formation of Invasive *Spartina* Hybrids

**DOI:** 10.3389/fpls.2019.00484

**Published:** 2019-04-16

**Authors:** Blanca Gallego-Tévar, María D. Infante-Izquierdo, Enrique Figueroa, Francisco J. J. Nieva, Adolfo F. Muñoz-Rodríguez, Brenda J. Grewell, Jesús M. Castillo

**Affiliations:** ^1^Departamento de Biología Vegetal y Ecología, Universidad de Sevilla, Seville, Spain; ^2^Departamento de Ciencias Integradas, Universidad de Huelva, Huelva, Spain; ^3^USDA-ARS Invasive Species and Pollinator Health Research Unit, Department of Plant Sciences, University of California, Davis, Davis, CA, United States

**Keywords:** global warming, alien species, pollination, seedling establishment, spatial distribution

## Abstract

Climate change can induce temporary, spatial or behavioral changes in species, so that only some species can adapt to the new climatic conditions. In the case of invasive species, it is expected that they will be promoted in a context of global change, given their high tolerance to environmental factors and phenotypic plasticity. Once in the invaded range, these species can hybridize with native species thus introducing their genotype in the native biota. However, the effects that climate change will have on this process of invasion by hybridization remain unclear. We evaluated the historical establishment of the reciprocal hybrids between the native *Spartina maritima* and the invasive *S. densiflora* in the Gulf of Cadiz (SW Iberian Peninsula) and we related it to climatic changes during the period 1955–2017. Our results showed that, according to their dating based on their rate of lateral expansion rates, the establishment of *S. maritima × densiflora* and *S. densiflora × maritima* in the Gulf of Cadiz has occurred in the last two centuries and has been related to changes in air temperature and rainfall during the flowering periods of their parental species, with antagonist impacts on both hybrids. Thus, the hybrid *S. densiflora × maritima* has been established in years with mild ends of spring and beginning of summer when the flowering of *S. maritima* lengthened and its pollen production was higher, and it coincided with the beginning of the flowering period of *S. densiflora*. Moreover, the establishment of this hybrid was related to higher spring/summer rainfalls, probably due to the reduction in salinity in middle marshes. However, the hybrid *S. maritima × densiflora*, was established mainly in warmer spring/summers in which the proportion of pollen:ovule of *S. maritima* was reduced favoring its pollination by *S. densiflora*. As a consequence of the promotion of *S. maritima × densiflora* with climate change, the native and endangered species *S. maritima* would be threatened, as both taxa share the same habitat and the hybrid shows a remarkably higher competitive potential.

## Introduction

New climatic conditions induced by global warming can lead to alterations of different biological processes in both plant and animal species (Parmesan, 2006). These changes include temporal, spatial or behavioral adjustments of species, such as changes in distribution range, phenological or physiological characteristics (Bellard et al., 2012). In this context, it is expected that many species will not be able to adapt to the new conditions, leading to a generalized increase of the extinction rate (Urban, 2015). In contrast, species with specific life-history traits that confer them high capacity to adapt to environmental changes, such as invasive species, are likely to be promoted in the new climatic scenario (Dukes and Mooney, 1999; Vilà et al., 2007).

Biological invasions and their drastic alteration of natural ecosystems are widespread and have long been recognized as a significant component of global environmental change (Vitousek et al., 1997, 2017). Understanding how environmental change factors are influencing fundamental biological processes is imperative for conservation and management of natural ecosystems. The continued spread of non-native plant species, synergistic with accelerating global environmental changes, poses significant challenges for understanding how natural ecosystems and the native plant communities will be altered (Drenovsky et al., 2012). Once introduced, one way by which invasive species displace or exclude native species is through hybridization and introgression (Rhymer and Simberloff, 1996), diluting or assimilating the native genotype (Huxel, 1999). Global change is expected to increase the likelihood of hybridization between native and invasive species (Muhlfeld et al., 2014), both by the increment in the introduction of species, and the changes in their ranges so that sympatry between divergent species may increase (Garroway et al., 2010; Hoffmann and Sgrò, 2011). However, studies on the biological processes underlying the increase in interspecific hybridization related to climate change are limited (Becker et al., 2013; Muhlfeld et al., 2014).

In this context of global environmental changes, many invasive plants are clonal organisms (Liu et al., 2006; Keser et al., 2014), and their longevity is an important demographic trait for understanding the life history, population dynamics, ecology and evolutionary fitness of plant species, (Harper, 1977; Schmid, 1990; Silvertown, 1991). Long-lived plant clones have been documented in various aquatic and wetland ecosystems (Santamaría, 2002). The asexual reproduction of clonal growth results in size expansion of genets and increased fitness as greater floral production in larger clones increases the potential for outcrossing and sexual reproduction (Barrett, 2015). Extreme longevity of clonal species allow genets to persist through periods with environmental conditions when sexual reproduction is rare or precluded. Clonality is a survival strategy of plant species that may support longevity at millennial timescales and through rapid global environmental changes (Bricker et al., 2018).

Cordgrasses (genus *Spartina*) are an adequate model for the study of hybridization between native and invasive species since there are different documented examples of interspecific hybridization after the introduction of one species in the native range of another (Strong and Ayres, 2013). These halophytic grasses, typical of tidal marshes, exhibit both sexual and clonal reproduction by rhizomes (Bortolus, 2006; Castillo and Figueroa, 2009a), forming clones that can remain for long periods of time (Castellanos et al., 1998; Travis and Hester, 2005). Asexual expansion by rhizomes is key for the colonization of surrounding areas around *Spartina* clones (Castillo and Figueroa, 2009a), whereas sexual reproduction by abundant seeds dispersed to short, medium and long distances allow them to colonize new salt marshes (Kittelson and Milton, 1997; Castillo et al., 2014). *Spartina* are anemophilous and protogynous species (Davis et al., 2004), whose flowering period occurs in the warm season (late spring-summer) and is controlled by environmental factors such as temperature or photoperiod (Ranwell, 1967; Seneca and Blum, 1984; Gray et al., 1991; Thompson, 1991). Previous studies on *Spartina alterniflora* Loisel., *Spartina anglica* C.E.Hubb., and *Spartina patens* (Aiton) Muhl., revealed that protogyny negatively affects autogamy (self-pollination) favoring cross-pollination (Bertness and Shumway, 1992; Fang et al., 2004; Li et al., 2008). However, Daehler (1998) indicated that protogyny was not enough to inhibit selfing in *S. alterniflora*. In the Gulf of Cadiz (Southwest Iberian Peninsula), native *Spartina maritima* (Curtis) Fernald and invasive *Spartina densiflora* Brongn. from the East Coast of South America have hybridized giving rise to two sterile reciprocal hybrids: *S. maritima × densiflora* has colonized low marshes and *S. densiflora × maritima* has invaded middle marshes (Castillo J.M. et al., 2010). According to the predictions of climate change models, these cordgrass populations will be subjected to increases in temperature and decreases in precipitation that will be more accentuated during the summer season (Anaya-Romero et al., 2015; IPCC, 2015). In order to know how these climatic changes are affecting the process of hybridization between native and invasive species, we carried out a study in which we dated different clones of *S. maritima × densiflora* and *S. densiflora × maritima* based on their size and lateral expansion rates in three estuaries of the Gulf of Cádiz. Then, their establishment dates were related to the historical data of rainfall and temperature. Our hypothesis was that the increase in temperature and decrease in rainfall associated with climate change would induce changes in the reproductive traits of *S. maritima* and *S. densiflora*, altering the process of hybridization between both species, since both parental species show high levels of phenotypic plasticity in response to changing environmental conditions (Castillo et al., 2005, 2014, 2018; Grewell et al., 2016).

## Materials and Methods

### Study Area and Taxa

**FIGURE 1:**
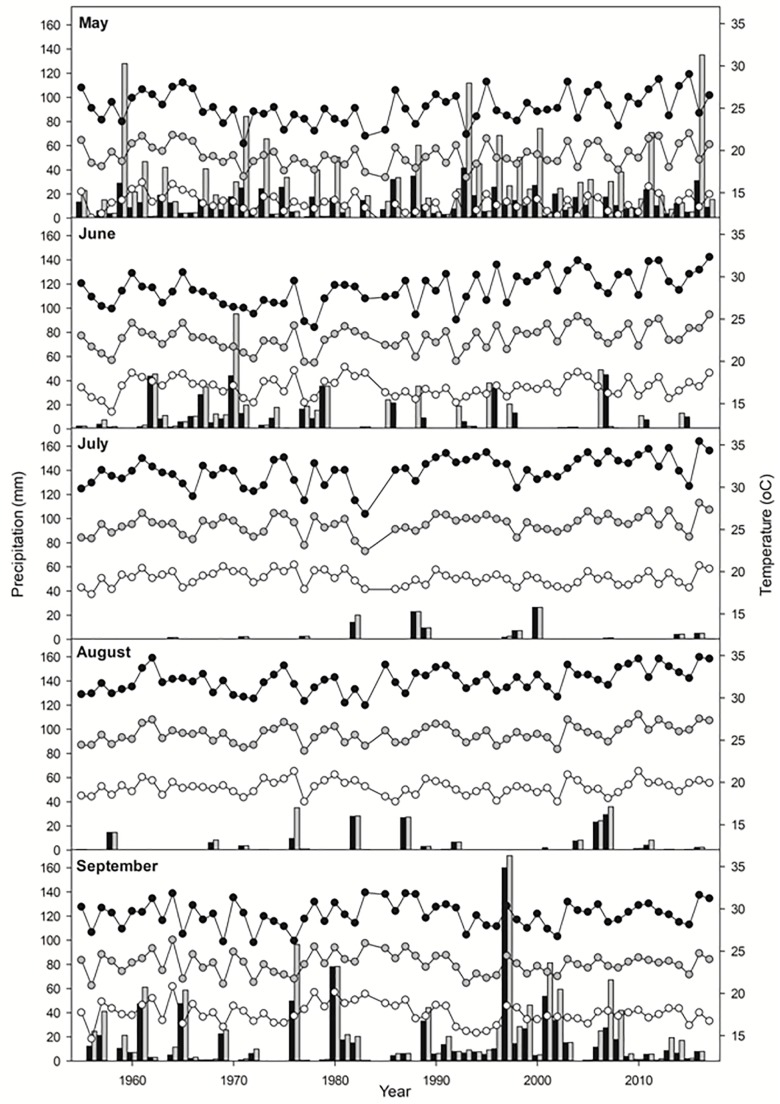
Meteorological data in May, June, July, August, and September from 1955 to 2017 in Southwest Iberian Peninsula. Mean monthly precipitation (black bars), maximum daily precipitation (gray bars), mean maximum temperatures (black circles), mean average temperatures (gray circles) and mean minimum temperatures (white circles).

This work was conducted in the estuaries of the rivers Tinto-Odiel (37° 08′–37° 20′ N; 6° 45′–7° 02′ W), Piedras (37° 12′–37° 18′ N; 7° 06′–7° 12′ W) and Guadiana (37° 10′–37° 16′ N; 7° 16′–7° 28′ W) along the Atlantic coast of Southwest Iberian Peninsula (Gulf of Cadiz). Tidal regime and vegetation for this area have been described by Castellanos et al. (1994), Figueroa et al. (2003); and Castillo et al. (2008). Salt marshes in these three estuaries are characterized by a vegetation zonation pattern in which low marshes (marsh elevation between +2.44 to +2.91 m Spanish Hidrographic Zero [SHZ]) are mainly occupied by *S. maritima*, *Salicornia ramosissima* J. Woods, and *Sarcocornia perennis* (Mill.) A.J. Scott., middle marshes (between +2.91 to +3.37 m SHZ) by *Atriplex portulacoides* (L.) Allen, the hybrid *Sarcocornia perennis × fruticosa* (Gallego-Tévar et al., 2018a) and invasive *Spartina densiflora*, and high marshes ( > +3.37 m SHZ) by *Arthrocnemum macrostachyum* (Moric.) C. Koch, *Suaeda vera* Forssk. ex J.F.Gmel., *Limoniastrum monopetalum* (L.) Boiss. and *Atriplex halimus* L. The mean average annual temperature for the period 1955–2017 is 18 ± 1°C, with a maximum temperature of 24 ± 1°C and a minimum of 13 ± 1°C (AEMET, 2018; CLIMA, 2018; Figure 1). Mean annual rainfall is 500 ± 150 mm, varying between 250 and 850 mm with 75–85 days of rain per year and 4–5 months of dried period around June and September (AEMET, 2018; CLIMA, 2018; Figure 1), when evapotranspiration leads to hypersalinity on the highest parts of the salt marshes (Castellanos et al., 1994). Storms events are generally frequent over the autumn and winter months with significant wave height values reaching up to 7 m (Plomaritis et al., 2015). Some predictions of climate change in this area indicate temperature increases of 2.8–6.1°C for minimum temperature and 3.3–7.2°C for maximum temperature, as well as a decrease of 12–32% rainfall by 2100, in winter-summer, respectively (Anaya-Romero et al., 2015).

Tussocks of the exotic hybrids between the native European cordgrass *S. maritima* (2*n* = 6x = 60) and the invasive *S. densiflora* (2*n* = 7x = 70) have been observed in these three estuaries. Two different reciprocal hybrids have been described, one whose seed parent is *S. densiflora* (*S. densiflora × maritima*, 2*n* = 6.5x = 65) that colonizes middle marshes where its maternal species is more frequent, and the other is *S. maritima × densiflora* (2*n* = 9.5x = 95, by unreduced gamete of *S. maritima*) whose habitat is low marshes as well as its seed parent *S. maritima*. These *Spartina* hybrids are particularly abundant in the Guadiana Estuary (hundreds) while they are very rare (<10 tussocks) in the Tinto-Odiel and Piedras Estuaries (Castillo J.M. et al., 2010). Both parental species are abundant in every location where the hybrids have established. *Spartina maritima* is more abundant at low elevations (low marshes, drainage channels and intertidal ponds at middle marshes) and *S. densiflora* typically forms dense stands in middle- high marshes, though it can also invade low marshes (Gallego-Tévar et al., 2018a). Both *Spartina* hybrids exhibit certain transgressive traits that confer hybrid vigor (Gallego-Tévar et al., 2018b). These hybrids invade throughout the entire elevation range of the intertidal gradient, from low to high marshes, where they are able to displace native species and alter the typical plant zonation pattern of these salt marshes (Gallego-Tévar et al., 2018a). At present, the current sterility of both reciprocal hybrids, *S. maritima × densiflora* and *S. densiflora × maritima*, limits their expansion, but fertility by allopolyploidization is known to be a frequent process in *Spartina* hybrids (Strong and Ayres, 2013). Both hybrids can be clearly differentiated in the field based on their phenotypes: *S. maritima × densiflora* shows less dense tussocks with shorter flowered tillers with more nodes and shorter leaves than *S. densiflora × maritima* (Supplementary Figures S1, S2). The flowering period for *S. maritima* has been described between May and July and for *S. densiflora* between June and December (Valdes et al., 1987). The tussocks of these hybrid modular plants were considered genets (clones) that were composed by units of rhizome and aerial shoots or tillers (ramets). Genets (*Spartina* clones) were clearly differentiated since they formed distinct circular tussocks separated from each other by unvegetated spaces or by cover of other halophyte species. Those few clones that did not present clear boundaries or were coalescing with adjacent clones were excluded from our study.

### Lateral Expansion Rate of *Spartina* Tussocks

Lateral expansion rates of hybrid tussocks were calculated as a preliminary step to estimate their age. Lateral expansion rates (cm yr^−1^) by rhizomes of the tussocks of both *Spartina* hybrids were calculated as the maximum diameter increment of individual tussocks in a given period; recording their diameter twice in different dates. For this purpose, the diameters of five tussocks of *S. densiflora × maritima* and 24 tussocks of *S. maritima × densiflora* were measured in Guadiana Estuary (37° 10′–37° 16′ N, 7° 16′–7° 28′ W) in February 2005. Also, the diameters of one tussock of *S. densiflora × maritima* and two tussocks of *S. maritima × densiflora* were recorded in Piedras Estuary (37° 12′–37° 18′ N, 7° 06′–7° 12′ W) in March 2005 (Supplementary Table S1). In order to compare the lateral expansion rates of both hybrids with their parental species, we also recorded the maximum diameter of 13 tussocks of *S. maritima* in January and September 2000 and of seven tussocks of *S. densiflora* in February 1997 and June 1999 at the East of Bacuta Island in Odiel Marshes (37° 13′ 41″ N; 6° 57′ 41″ W), and of other 10 tussocks of *S. densiflora* in October 1996 and September 2000 at the left bank of the main channel of the Tinto-Odiel Estuary (37° 13′ 32″ N; 6° 57′ 06″ W). All measured tussocks were chosen randomly in each sampled population. All the maximum diameters of the individuals of both hybrids and both parental species were remeasured in May 2018.

### Estimation of *Spartina* Hybrids Age

The age of each hybrid *Spartina* tussock was estimated to relate their establishment period with changing meteorological conditions. The maximum diameter of tussocks of *S. maritima × densiflora* were recorded in the estuaries of Tinto-Odiel (*n* = 3 tussocks; half of the 6 known tussocks in this estuary), Piedras (*n* = 4 tussocks; all known tussock of this hybrid in this estuary) and Guadiana (*n* = 18 tussocks; all known tussocks recorded at the north of San Bruno Marsh [37°10′–37°16′N, 7°28′–7°16′W]) in May 2018. The maximum diameter of *S. densiflora × maritima* tussocks was measured in the estuaries of Tinto-Odiel (*n* = 2 tussocks; all known tussock of this hybrid in this estuary), Piedras (*n* = 1 tussocks; all known tussock of this hybrid in this estuary) and Guadiana (*n* = 205 tussocks; all known tussocks recorded at the north of San Bruno Marsh) in May 2018. The seed parent of measured hybrids in Tinto-Odiel and Piedras estuaries were identified by Castillo J.M. et al. (2010). In Guadiana, measured *Spartina* hybrids at the low marshes were identified as *S. maritima × densiflora* and those at middle marshes as *S. densiflora × maritima* following Castillo J.M. et al. (2010).

The ages (in yr) of individual tussocks of both *Spartina* hybrids were estimated as the ratio between the tussock maximum diameter (cm) and its lateral expansion rate (cm yr^−1^). Lateral expansion rate by rhizomes was recorded during periods longer than 10 years for both hybrids, integrating the environmental variability that hybrid tussocks were exposed to during significant periods of their actual life span. Our growth model considered that recorded integrated lateral expansion rates were constant during the whole life span of the studied tussocks, resulting in constant radial growth of the clone for uncrowded, density independent individual plants. In this sense, Dennis et al. (2011) showed that factors intrinsic to *Spartina* tussocks dominated the effects of large scale abiotic factors on clone growth, resulting in constant radial growth over time. As in our study, previous works have estimated the age of clonal herbs also using the size of the genet and its growth rate (de Witte and Stöcklin, 2010).

### Relationships Between *Spartina* Hybrids Establishment and Meteorological Conditions

Relationships between changing meteorological conditions and the establishment of *Spartina* hybrids were recorded to analyze to effects of climate change on the invasion of hybrids. The year of establishment of every hybrid tussock was inferred from its estimated age. Then, the annual number of established tussocks for every *Spartina* hybrid (*S. maritima × densiflora*, *N* = 25 tussocks; *S. densiflora × maritima*, *N* = 208 tussocks) was related to the meteorological conditions of every year of establishment. Meteorological monthly data (mean rainfall, maximum daily rainfall, mean of minimum, average and maximum temperatures) from 1955 to 2017 were obtained from the meteorological station of the city of Huelva (37°15′35.02″N, 6°56′55.37″W). This period of meteorological data is the maximum available in the Spanish State Meteorological Agency (AEMET, 2018). Specifically, monthly meteorological data for May, June, July, August, and September were used in our analyses since these are the months in which the flowering and fruiting time of *S. maritima* (May–September) and *S. densiflora* (June–December) may coincide in the Gulf of Cadiz (Valdes et al., 1987).

Additionally, we studied the genet dynamics of the historical establishment of *Spartina* hybrids in a model marsh known locally as San Bruno (Guadiana Estuary), where the greatest density of hybrids (hundreds) has been observed. With this aim, the diameter of each tussock and its spatial distribution (using a Garmin Oregon 550t decametric GPS [Garmin Ltd., KA, United States]) was recorded for both *Spartina* hybrids in a total area of 28 ha in May 2018. The year of establishment of each hybrid tussock was estimated as reported above. Hybrid tussocks were classified according to their decade of establishment and represented on the corresponding aerial color photograph for each decade obtained from those available for the period 1943–2018 in the photo libraries of the Spanish National Geographical Institute (IGN, 2018) and the Andalusian Institute of Statistics and Cartography (IECA, 2018).

### Flowering Period and Pollen:Ovule Ratio of *Spartina maritima*

Flowering phenology and pollen:ovule ratio of *S. maritima* were studied since previous field observations suggested they may change considerably between years depending on changing environmental conditions. The duration of the flowering period of *S. maritima* was evaluated for 20 tussocks during a warm flowering period in 2017 (mean temperature 24.1 ± 1.7°C and maximum temperature 30.6 ± 2.1°C for May–July) and for five tussocks during a mild flowering period in 2018 (mean temperature 21.4 ± 1.6°C and maximum temperature 27.2 ± 1.7°C for May–July) in Odiel Marshes. Information on the duration of the flowering period of *S. densiflora* was obtained from Valdes et al. (1987) and Castillo and Figueroa (2009a).

The number of pollen grains per anther of *S. maritima* was calculating by extracting five anthers from each of two tussocks in two different locations in July 2017, staining them in a mix of a few drops of cotton blue lactophenol solution in 1.5 ml of water, taking three aliquots of 10 μl and counting pollen grains on a microscope slide. The total number of pollen grains per anther was calculated as product of the mean pollen concentration in the aliquots (*n* = 3) per the total volume of pollen suspension. The number of pollen grains per anther has been found to be constant in the Poaceae family (Prieto-Baena et al., 2003). Subsequently, the calculated value of pollen grains per anther was used for the calculation of the pollen:ovule ratio of *S. maritima* in 25 spikelets chosen randomly from each of 20 inflorescences collected at random from five tussocks in two locations in Odiel Marshes in July 2017 (warm flowering period) and July 2018 (mild flowering period). For this propose the number of spikelets with exerted stamens and the number of spikelets per inflorescence were counted during anthesis. Then, the number of exerted stamens per inflorescence was estimated as product of the number of spikelets with exerted stamens per the number of spikelets per inflorescence and by three stamens per spikelet. The number of pollen grains per inflorescence was calculated for 2017 and 2018 as the product of the number of pollen grains per anther and the number of exerted stamens per inflorescence. Finally, pollen:ovule ratio was calculated as the quotient of the number of pollen grains per inflorescence and the number of spikelets per inflorescence, since the *Spartina* Genus presents an uniovular carpel and the number of seminal primordia per inflorescence corresponded to the number of spikelets on each inflorescence.

### Statistical Analyses

All the analyses were applied with a significance level (α) of 0.05 and they were conducted using the software Sigma-Plot for Windows (version 12.0, Systat Software Inc., IL, United States). Data series were verified for normality with the Shapiro-Wilk’s test and for homoscedasticity with Levene’s test, before the application of parametric analyses. In cases when data transformations (inverse, square root, or logarithm) were insufficient to meet assumptions of the parametric models, non-parametric tests were conducted. The lateral expansion rates of *S. densiflora*, *S. maritima* and their hybrids were compared using one-way analysis of variance (ANOVA) on ranks, using taxa as grouping factor and Dunn’s test as *post hoc* analysis. Diameter and estimated age of both *Spartina* hybrids were compared using Mann-Whitney *U*-test and Student’s *t*-test for impendent samples, respectively. Pollen:ovule ratio for *S. maritima* was compared between 2017 and 2018 using Student’s *t*-test for paired samples. To explore the relationships between the establishment of both *Spartina* hybrids and meteorological conditions, linear correlations (Pearson coefficient) between the numbers of annually established hybrids and monthly meteorological variables, and linear regressions between the numbers of annually established hybrids and mean maximum temperature were carried out. When the number of annually established hybrids was correlated with two or more meteorological variables, multiple regression analysis was carried out to explore relative weights (β).

## Results

The lateral expansion rate of *Spartina maritima × densiflora* (21 ± 2 cm yr^−1^) was not significantly different from that of its seed parent *S. maritima* (44 ± 6 cm yr^−1^), and its rate was higher than that of *S. densiflora × maritima* (4 ± 2 cm yr^−1^) which presented a similar lateral expansion rate to its seed parent *S. densiflora* (5 ± 0.3 cm yr^−1^) (Kruskal-Wallis, *H* = 47.421, *P* < 0.001; Dunn’s test, *P* < 0.05) (Supplementary Table S1 and Figure 1).

**FIGURE 2:**
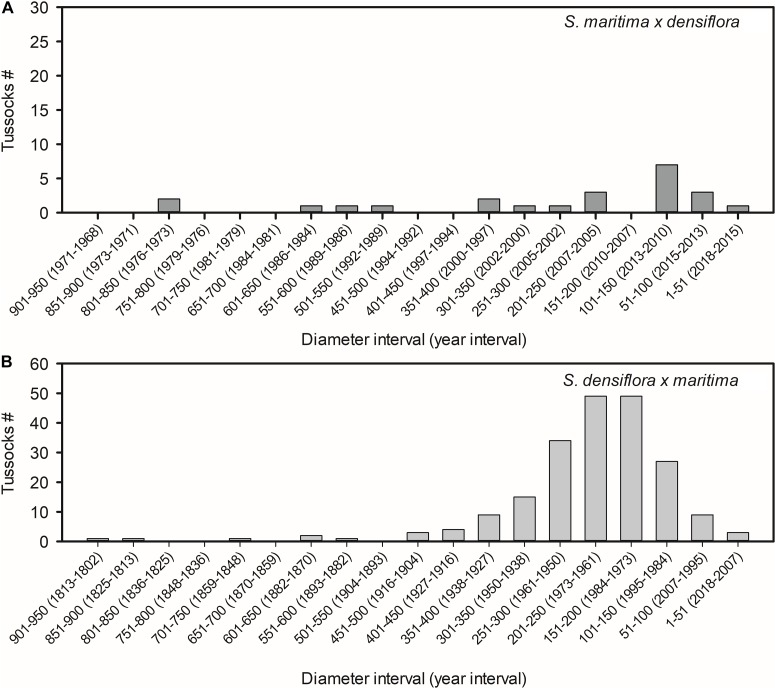
Number of tussocks for different size classes (diameters in cm) of the hybrids **(A)** *Spartina maritima × densiflora* (*n* = 22) and **(B)** *S. densiflora × maritima* (*n* = 208) in the estuaries of the rivers Tinto-Odiel, Piedras, and Guadiana (Southwest Iberian Peninsula). The years of establishment estimated according to their lateral expansion rates by rhizomes are indicated in parentheses.

Tussock diameters of *S. maritima × densiflora* and *S. densiflora × maritima* were 314 ± 55 cm and 238 ± 9 cm, respectively (Mann-Whitney test, *U* = 2284, *P* > 0.05). Accordingly, with their lateral expansion rates, those diameters corresponded to their establishment taking place 17 ± 3 year ago for *S. maritima × densiflora* and 54 ± 2 year ago for *S. densiflora × maritima* (*t*-test, *t* = −6.204, *P* < 0.001). The estimated date of establishment of the oldest tussock of *S. maritima × densiflora* was 1974 and the youngest was established in 2016 (Figure 2A), while the oldest and the youngest tussocks of *S. densiflora × maritima* were established in 1813 and 2007, respectively (Figure 2B).

Mean average and maximum temperatures in June, July, and August increased over time in the period 1955–2017 (Pearson correlation, *P* < 0.01). The highest monthly average and maximum temperatures were recorded in 2017 for June, in 2015 for July and in 2016 for August (Figure 1). However, mean average and maximum rainfall did not show a significant relationship with time for the aforementioned period (Pearson correlation, *P* > 0.05). For June, the highest average rainfall (two times greater than the next higher value) was registered in 1970, with the previous and following years being more humid than the average, while in seven of the last 13 years there was no precipitation. July and August were generally very dry, with some years reaching precipitations of 25 and 35 mm, respectively (Figure 1).

**FIGURE 3:**
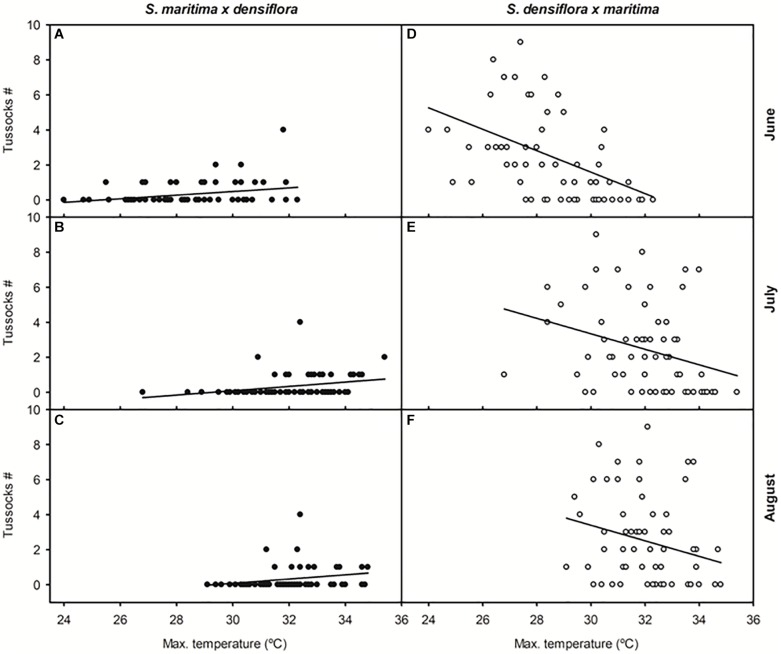
Relationships between the number of annually established tussocks of *Spartina* hybrids and mean maximum temperatures from 1955 to 2017 on the Gulf of Cadiz (Southwest Iberian Peninsula). Taxa: *Spartina maritima × densiflora* (black symbols); *S. densiflora × maritima* (white symbols). Months: **(A,D)** June, **(B,E)** July, and **(C,F)** August. Regression equations (*N* = 61–62): **(A)** *y* = –2.612 + 0.103x, *R* = 0.28, *P* < 0.05; **(B)** *y* = –3.605 + 0.123x, *R* = 0.30, *P* < 0.05; **(C)** *y* = –3.537 + 0.121x, *R* = 0.25, *P* = 0.05; **(D)** *y* = 19.957 – 0.612x, *R* = 0.47, *P* < 0.0001; **(E)** *y* = 16.617 + 0.442x, *R* = 0.30, *P* < 0.05; **(F)** *y* = 16.717 + 0.444x, *R* = 0.25, *P* < 0.05.

The number of recorded annually established tussocks of *S. maritima × densiflora* between 1955 and 2017 was positively correlated with mean maximum temperature in June, mean average and maximum temperatures in July (β = +1.219 and −0.449, respectively), and mean average temperature in August (Table 1 and Figure 3). On the contrary, the annual number of recorded established tussocks of *S. densiflora × maritima* was negatively correlated with mean average and maximum temperatures in June and mean maximum temperature in July and August (Table 1 and Figure 3). In all cases, the slopes of the negative regressions between the numbers of established tussocks of *S. densiflora × maritima* and maximum monthly temperatures were higher than the positives slopes for *S. maritima × densiflora* (Figure 3). Additionally, the annual number of established tussocks of *S. densiflora × maritima* increased together with mean average and maximum rainfall in June. Rainfall accounted for less weight in the model than air temperatures (average temperature: β = +0.917; maximum temperature: β = −1.396; average rainfall: β = +0.326; maximum rainfall: β = 0.373). Moreover, both *Spartina* hybrids showed a negative correlation between them regarding the annual number of established tussocks (Table 1). No correlation was found between the number of established hybrids and any of the monthly meteorological parameters in May or in September (Table 1).

**Table 1:** Correlations between the number of annually established of tussocks of *Spartina* hybrids and meteorological conditions.

	Max. Rainfall	Rainfall	Min T^*a*^	Average T^*a*^	Max T^*a*^
**May**					
Tussocks # (*Smxd*)	0.147	0.164	0.148	0.213	0.217
	0.255	0.204	0.252	0.097	0.090
Tussocks # (*Sdxm*)	0.016	0.005	0.136	−0.074	−0.181
	0.904	0.972	0.290	0.569	0.158
**June**					
Tussocks # (*Smxd*)	−0.071	−0.117	0.012	0.205	**0.283**
	0.588	0.369	0.924	0.110	**0.026**
Tussocks # (*Sdxm*)	**0.321**	**0.310**	−0.001	−**0.330**	−**0.471**
	**0.012**	**0.015**	0.992	**0.009**	**0.000**
**July**					
Tussocks # (*Smxd*)	0.210	0.189	0.068	**0.264**	**0.300**
	0.101	0.142	0.598	**0.038**	**0.018**
Tussocks # (*Sdxm*)	−0.025	−0.001	0.215	−0.155	−**0.302**
	0.846	0.995	0.094	0.230	**0.017**
**August**					
Tussocks # (*Smxd*)	0.055	0.070	0.176	**0.260**	0.246
	0.669	0.594	0.171	**0.041**	0.054
Tussocks # (*Sdxm*)	0.022	0.001	0.046	−0.165	−**0.253**
	0.869	0.997	0.723	0.200	**0.047**
**September**					
Tussocks # (*Smxd*)	−0.052	−0.056	−0.131	0.001	0.100
	0.688	0.665	0.310	0.992	0.437
Tussocks # (*Sdxm*)	−0.082	−0.116	0.242	0.090	−0.047
	0.525	0.369	0.058	0.486	0.717

Tussocks # (*Sdxm*)					

Tussocks # (*Smxd*)	−**0.289**				
	**0.023**				

*Pearson correlation coefficient (upper digit) and P-values (lower digit). Taxa: Spartina maritima × densiflora (Smxd); Spartina densiflora × maritima (Sdxm). Meteorological parameters: mean monthly maximum and average rainfall, and mean minimum, average and maximum temperature (T^a^) in Southwest Iberian Peninsula in May, June, July, and August from 1955 to 2017. N = 61–62. Significant correlations are marked in bold.*

**FIGURE 4:**
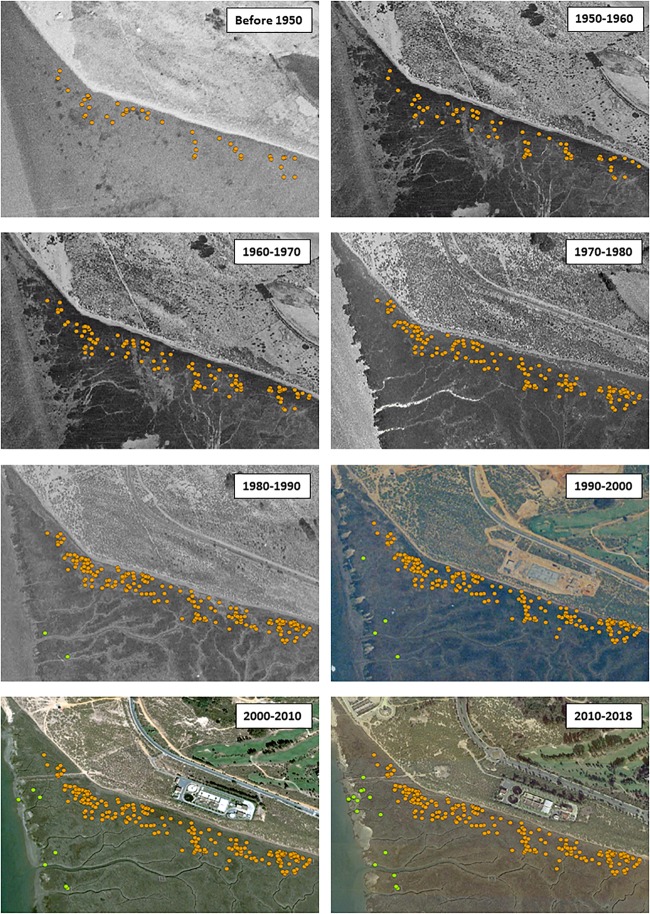
Historical spatial distribution of the hybrids *Spartina maritima × densiflora* (green symbols) and *S. densiflora × maritima* (orange symbols) in San Bruno Marsh (Guadiana Estuary, Southwest Iberian Peninsula), before 1950, 1950–1960 (aerial photography of 1956), 1960–1970 (aerial photography of 1956), 1970–1980 (aerial photography of 1973), 1980–1990 (aerial photography of 1984), 1990–2000 (aerial photography of 1996), 2000–2010 (aerial photography of 2005), and 2010–2018 (aerial photography of 2017).

In the model marsh of San Bruno (Guadiana Estuary), the number of tussocks identified as *S. densiflora × maritima* in middle marshes (205 tussocks) was much higher than that of *S. maritima × densiflora* in low marshes (18 tussocks). According to the estimated ages of these tussocks, the first hybrid to be established was *S. densiflora × maritima* (ca. year 1813) while the first tussock of *S. maritima × densiflora* did not appear until 1984. Since 2007, no novel tussocks of *S. densiflora × maritima* have been established, while the latest tussock of *S. maritima × densiflora* dates back to 2016 (Figure 4).

**FIGURE 5:**
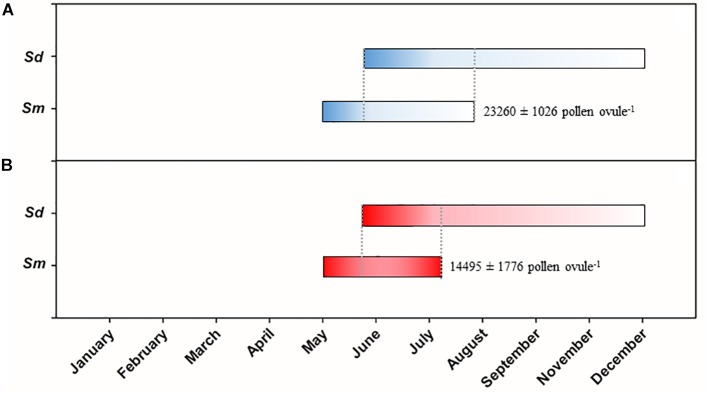
Flowering periods of native *Spartina maritima* (*Sm*) and invasive *S. densiflora* (*Sd*) in **(A)** 2018 (a mild flowering period) and **(B)** 2017 (a warm flowering period). Darker color represents the predominance of pistils in the inflorescence and lighter color is the predominance of stamens. The pollen:ovule ratio of *S. maritima* is indicated nearby its flowering bars. The flowering period of *S. densiflora* was obtained from Valdes et al. (1987) and Castillo and Figueroa (2009a).

The flowering period of *S. densiflora* extended from June to December following historical records by Valdes et al. (1987) and Castillo and Figueroa (2009a) (Figure 5). *S. maritima* started flowering at the beginning of May in both a very warm and a mild end of spring and beginning of summer in 2017 and 2018, respectively. The end of the flowering period of *S. maritima* was advanced in ca. 20 days in July in 2017 in comparison to July 2018 due to an abrupt end of its flowering. This provoked that many *S. maritima* flowers that had exposed their pistil did not exert their stamens. Thus, the pollen:ovule ratio during the flowering period of *S. maritima* was much lower in 2017 (14495 ± 1776 pollen ovule^−1^) than in 2018 (23260 ± 1026 pollen ovule^−1^) (paired Student *t*-test, *t* = 8.472, *P* < 0.001, df = 4) (Figure 5).

## Discussion

Our study focused on the dynamics of establishment of the reciprocal hybrids between native *Spartina maritima* and invasive *S. densiflora* provides new insights on the effects of climate change on the interactions between native and invasive species and on the origin of exotic hybrid plants.

The lateral expansion rates of the hybrids *Spartina maritima × densiflora* and *S. densiflora × maritima* were different from each other and similar to those of their corresponding seed parents, revealing the maternal influence on this plant trait. Maternal effect is a relevant process in gene expression of hybrids (Videvall et al., 2016) such as for growth characteristics of *Seneceio jacobea × aquaticus* (Kirk et al., 2005) or the hybrids between transgenic *Brassica napus* L. and wild *B. juncea* (L.) Vassilii Matveievitch (Di et al., 2009). For the reciprocal hybrids *S. maritima × densiflora* and *S. densiflora × maritima*, maternal effects have been previously observed in their response to different salinities (Gallego-Tévar et al., 2018b). The recorded greater expansion rate of native *S. maritima* was consistent with its growth form in “guerrilla” (*sensu* Lovett Doust and Lovett Doust, 1982) in which asexual lateral spreading by long rhizomes predominates over sexual reproduction (Castellanos et al., 1994). In contrast, invasive caespitose *S. densiflora* exhibited short rhizomes, growing in “phalanx” (*sensu* Lovett Doust and Lovett Doust, 1982) and forming dense tussocks (Castillo J. et al., 2010), with its dispersion occurring mainly by seeds (Kittelson and Milton, 1997). The lateral expansion rate recorded in this study for *S. maritima* was in the range of that reported previously by Figueroa et al. (2003) (38 cm yr^−1^) and by Castillo and Figueroa (2009b) (26 cm yr^−1^) in Odiel Marshes. Similarly, the recorded lateral expansion rate for *S. densiflora* was comparable to those reported by Kittelson and Milton (1997) and Nieva et al. (2005) (ca. 8 cm yr^−1^). The lateral expansion rates of both hybrids may be explained by their growth forms in relation to their parental species. Thus, *S. maritima × densiflora* expanded laterally more rapidly and with longer rhizomes than *S. densiflora × maritima*. Both hybrids formed dense turfs of tillers within their tussocks like *S. maritima*, but more clearly differentiated and of bigger sizes, so that the turfs of tillers within hybrid tussocks presented similar growth form than individuals tussocks of *S. densiflora*, but with taller tillers (Supplementary Figure S2; Gallego-Tévar, personal observation). The bare spaces within the hybrid turfs usually constituted intertidal ponds or were occupied by other halophytes (Supplementary Figure S2; Gallego-Tévar, personal observation), while the turfs of tillers within tussocks were rarely colonized by other halophytes (Gallego-Tévar et al., 2018a).

As a consequence of the greater lateral expansion rate of *S. maritima × densiflora* in relation to *S. densiflora × maritima*, its tussocks were dated younger, despite having both hybrids similar sizes. When the number of annually established hybrid tussocks (1955–2017), according to their estimated ages, was related to meteorological conditions, correlations were found for both hybrids with mean maximum monthly temperature in June, July and August. These were the only months for which significant increases in temperatures were registered in the analyzed time period, being also these months the common period of flowering for both parental species (Valdes et al., 1987). The above-mentioned increase in air temperature during the end of spring and the beginning of summer was consistent with the predictions of climate change models for the Mediterranean region (Giorgi and Lionello, 2008; Giannakopoulos et al., 2009; Kovats et al., 2014). The correlations between the number of established tussocks and mean maximum monthly temperatures were positive for *S. maritima × densiflora* and negative for *S. densiflora × maritima*. Therefore, an alternation in the establishment of both hybrids in relation to changing climatic conditions was observed, so that *S. maritima × densiflora* establishment increased and *S. densiflora × maritima* decreased in warmer spring-summers, and *vice versa*. Even with an error interval of 1 year in our dating of hybrid tussocks, their establishment would be related to atmospheric temperatures since they, in general, tended to increase more than most of the recorded interannual differences.

The effects of meteorological conditions (and climatic conditions in the long term) on the formation of each of the *Spartina* hybrids may be related to alterations in the flowering dynamic of *S. maritima* phenology. Air temperature is an important factor in the induction of flowering in grasses (Cooper and Calder, 1964; Heide and Heide, 1994). Both *S. maritima* and *S. densiflora* flowers during the end of spring and the beginning of summer when increasing temperatures and photoperiods co-occur and both cordgrasses are protogynous, with the pistil emerging earlier than the stamens starting from upper flowers in the inflorescences to their bottom (Davis et al., 2004). In mild years during May–July, as those frequently recorded in the first decades of the twentieth century, *S. maritima* lengthened its flowering, starting in May and being prolonged until the end of July, and *S. densiflora* started its flowering in July. In these meteorological conditions similar to those of 2018, *S. maritima* finished its flowering exerting all its stamens, showing high pollen:ovule ratio. Thus, it would be expected that the hybrid of which *S. densiflora* is the seed parent (*S. densiflora × maritima*) would be the most abundant, as recorded in our study. There would be a greater chance of matching the last stamens (pollen) of *S. maritima* to the first pistils (stigma) of *S. densiflora*, when its stamens have not yet been exerted, than *vice versa*. In contrast, during warm flowering periods, as those recorded mainly at the end of the twentieth century and the beginning of the twenty-first century as 2017, the flowering of *S. maritima* finished early and abruptly in July so that the stamens of some inflorescences did not get to be exerted while the pistils were already exerted, which was reflected in low pollen:ovules ratio for *S. maritima*. Thus, the result of low pollen:ovule ratio of *S. maritima* during warm flowering periods would result, as recorded in our study, in a higher formation of the hybrid *S. maritima × densiflora*. On the other hand, the broad flowering period of *S. densiflora* from June to December reported by Valdes et al. (1987) and Castillo and Figueroa (2009a) in the Gulf of Cadiz and (Bortolus, 2006) in different regions worldwide would be related to the high phenotypic plasticity of this cordgrass in response to contrasted environmental conditions (Nieva et al., 2001; Castillo et al., 2014, 2018; Grewell et al., 2016). Seneca and Blum (1984) observed that air temperature was the main factor controlling the flowering of *Spartina alterniflora* Loisel., blooming at 22–26°C, while *Spartina foliosa* Trin. flowered in a wider temperature range being less dependent on this factor. This increase in the flowering of *S. alterniflora* with temperature was mainly associated to high carbon assimilation, although photoperiod-temperature direct induction was not ruled out. Also, mild temperatures in spring and early summer are known to delay flowering of the allopolyploid *Spartina anglica* C.E.Hubb. (Ranwell, 1967; Gray et al., 1991; Thompson, 1991). Another factor that would favor the formation of the hybrid *S. maritima × densiflora* at low marshes is the fact that *S. densiflora* in this stressful habitat flowers normally earlier than at middle marshes (Nieva et al., 2005), facilitating the pollination of *S. maritima*.

The number of annually established tussocks of *S. densiflora × maritima* was also higher in the years with higher rainfall in June. Published accounts on the effects of climate change on flowering phenology have found no direct relationships between high rainfalls and change in flowering of grasses (Cleland et al., 2006; Sherry et al., 2007), including cordgrasses such as *S. alterniflora* and *Spartina patens* (Aiton) Muhl. (Charles and Dukes, 2009). However, an increase in rainfall during the beginning of the summer would lead to a reduction in soil salinity in salt marshes (Burdick et al., 2001), which would favor the development of *S. densiflora* (Castillo et al., 2005, 2014, 2018; Grewell et al., 2016) and *S. maritima* (Naidoo et al., 2012). High salinities can reduce flowering of halophytes (Flowers et al., 1986; Ventura et al., 2014). In this sense, *S. densiflora* and *S. foliosa* from San Francisco Bay reduced their production of inflorescences at hypersalinity in relation to lower salinities (Gallego-Tévar et al., unpublished data). In our study, rainfall was only related to the establishment of the hybrid *S. densiflora × maritima* in middle marshes, which is consistent with the fact that the highest salinities during the dry season (summer) are reached in middle marshes (Contreras-Cruzado et al., 2017). Thus, according to our results, *S. densiflora × maritima* hybrids were formed mostly between 1961–1984 coinciding with the years of lower maximum temperatures and higher rainfall of the period 1955–2017. However, changing flowering phenology of the parental species seemed to be the process driving the switch in the appearance of studied reciprocal hybrids. The influence of environmental conditions, such as salinity, on the establishment of these hybrids cannot be ruled out as factor determining their abundance (Noe and Zedler, 2001). In this sense, Gallego-Tévar et al. (2018a) revealed that studied *Spartina* hybrids colonized sediments with lower salinities than their parental species. Therefore, an increase in sediment salinity due to higher evapotranspiration and sea level raise combined with reduced rainfall could limit the establishment of the hybrids. Nevertheless, both studied hybrids show higher fitness than their parents at high salinities due to transgressive traits as adult plants (Gallego-Tévar et al., 2018b), which could support their growth and their competitive ability in future climate change scenarios.

Climate change models predict increases in maximum temperatures and decreases in rainfall in the Mediterranean basin especially in summer (Giorgi and Lionello, 2008; Giannakopoulos et al., 2009; Kovats et al., 2014; IPCC, 2015), so we predict a reduction of the formation of the hybrid *S. densiflora × maritima* in favor of *S. maritima × densiflora*. In view of our results, the rise in just a few degrees in air maximum temperature during the flowering periods of *S. maritima* and *S. densiflora* increases the probabilities of establishment of the hybrid *S. maritima × densiflora* in relation to *S. densiflora × maritima*. *S. maritima × densiflora* was formed mainly with mean maximum temperature in June higher than 29°C, whereas *S. densiflora × maritima* with mean maximum temperature in June lower than 31°C. The predictions of maximum temperature increase with climate change in summer in Southern Iberian Peninsula range from +2.5°C in 2040 to +7.2°C in 2100 (Anaya-Romero et al., 2015), so a marked intensification in the maternal switch in the formation of *Spartina* hybrids is expected. Specifically in Southwest Iberian Peninsula where this study was carried out, atmospheric temperatures and the duration and intensity of heat waves are increasing, and are predicted to continue to increase in the mid-term (2046–2065) and specially in the long-term (2081–2100) future due to climate change (Pereira et al., 2017). Thus, if the current trend continues, most summer days would present maximum daily temperatures higher than 30–35°C by the end of the XXI century (Pereira et al., 2017). Under these future conditions, our results suggest only the invasive hybrid *S. maritima × densiflora* may establish and persist.

The increase of the appearance of *S. maritima × densiflora* with climate change would pose a greater threat to the native species *S. maritima*, since this hybrid grows rapider, taller and in the same habitat than its seed parent (Castillo J.M. et al., 2010). This competitive pressure would be added to the one exerted by sea level rise on low marsh halophytes (Schile et al., 2014) such as *S. maritima*, which is included in different European red lists as that one from the South of Iberian Peninsula (Cabezudo et al., 2005).

## Author Contributions

BG-T, MI-I, EF, AM-R, and JC contributed to the idea, topic, background information, and experiment planning. BG-T, MI-I, FN, AM-R, and JC carried out the field work and analyzed the data. BG-T, BG, and JC wrote the manuscript.

## Conflict of Interest Statement

The authors declare that the research was conducted in the absence of any commercial or financial relationships that could be construed as a potential conflict of interest.
