# Supplementary material for: Some Like It Hot: Maternal-Switching With Climate Change Modifies Formation of Invasive Spartina Hybrids
Source: Front Plant Sci. 2019 Apr 16;10:484. doi: 10.3389/fpls.2019.00484 (PMC6477182; doi:10.3389/fpls.2019.00484)
Supplement: Supplementary file 1 [file Table_1.pdf]

Supplementary Material

1 Supplementary Figures and Tables

Supplementary Table 1. Tussocks of *Spartina* hybrids used for the calculation of the lateral expansion rate by rhizomes, indicating their estuary, the main accompanying species, the date for the first diameter measure (the diameter of all tussocks was re-measured on 17-18 May 2018), and their lateral expansion rate (cm yr^{-1}). Species: *Sd* = *Spartina densiflora*; *Sm* = *Spartina maritima*; *Sp* = *Sarcocornia perennis*; *Sf* = *Sarcocornia fruticosa*; *Ap* = *Atriplex portulacoides*; *Spxf* = *Sarcocornia* hybrid between *S. perennis* and *S. fruticosa*.

Spartina densiflora x maritima				*Spartina maritima x densiflora*			
**Estuary**	**Main accompanying species**	**Date of 1st measurement**	**Lateral expansion rate**	**Estuary**	**Main accompanying species**	**Date of 1st measurement**	**Lateral expansion rate**
Piedras	*Sd*	04/03/2005	12	Piedras	*Spxf*	04/03/2005	43
Guadiana	*Sd*	20/01/2003	3	Piedras	*Spxf*	04/03/2005	54
Guadiana	*Sd*	20/01/2003	2	Guadiana	*Sm*	15/02/2006	27
Guadiana	*Sd*	20/01/2003	4	Guadiana	*Sp*	15/02/2006	17
Guadiana	*Sd*	20/01/2003	1	Guadiana	*Sp*	15/02/2006	18
Guadiana	*Sd*	20/01/2003	3	Guadiana	*Sp*	15/02/2006	21
				Guadiana	*Sp*	15/02/2006	16
		**Mean ± SEM**	**4 ± 2**	Guadiana	*Sp*	15/02/2006	19
				Guadiana	*Sf*	15/02/2006	25
				Guadiana	*Sf*	15/02/2006	25
				Guadiana	*Sf*	15/02/2006	35
				Guadiana	*Sf*	15/02/2006	34
				Guadiana	*Ap*	15/02/2006	23
				Guadiana	*Ap*	15/02/2006	21
				Guadiana	*Ap*	15/02/2006	15
				Guadiana	*Ap*	15/02/2006	18
				Guadiana	*Ap*	15/02/2006	25
				Guadiana	*Sd*	15/02/2006	9
				Guadiana	*Sd*	15/02/2006	15
				Guadiana	*Sd*	15/02/2006	8
				Guadiana	*Sd*	15/02/2006	6
				Guadiana	*Spxf*	15/02/2006	6
				Guadiana	*Spxf*	15/02/2006	19
				Guadiana	*Spxf*	15/02/2006	10
				Guadiana	*Spxf*	15/02/2006	25
				Guadiana	*Spxf*	15/02/2006	27
						**Mean ± SEM**	**21 ± 2**

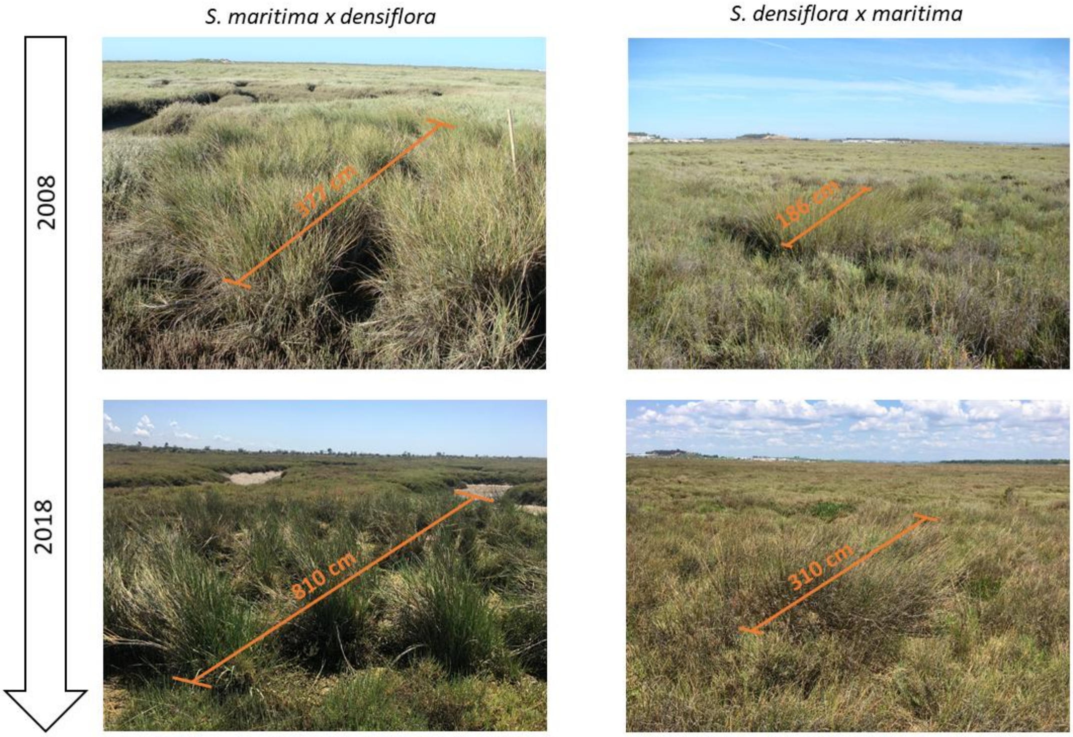

Supplementary Figure 1. Size differences of one individual of *S. maritima x densiflora* and one of *S. densiflora x maritima* from Piedras Estuary between 2008 and 2018. The diameters of each individual and year are indicated in each picture. Images are not scaled.

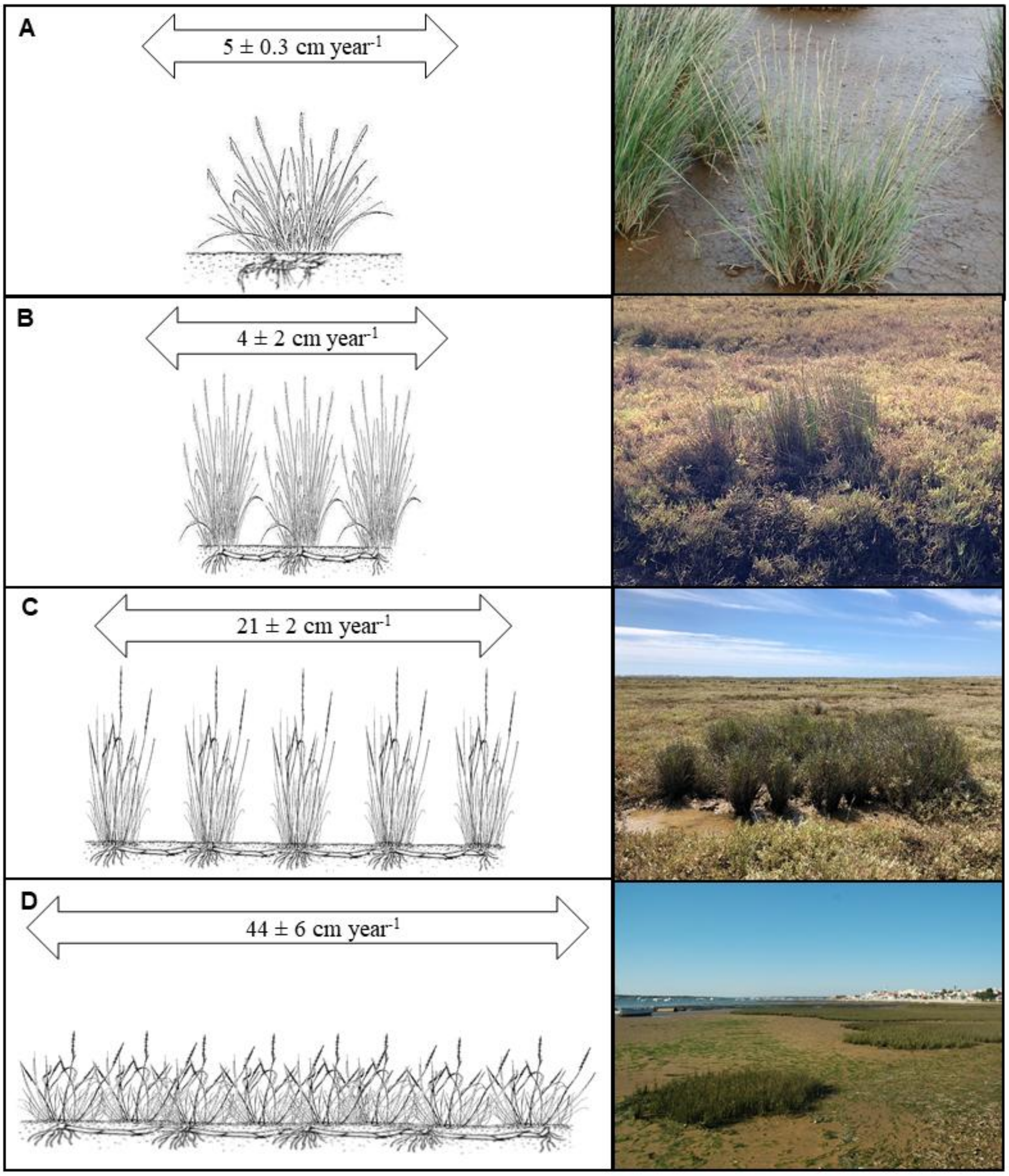

Supplementary Figure 2. Schematic models of growth forms, field pictures and lateral expansion rates by rhizomes for tussocks of **(A)** *Spartina densiflora* (n = 17), **(B)** *S. densiflora x maritima* (n = 6), **(C)** *S. maritima x densiflora* (n = 26) and **(D)** *S. maritima* (n = 13).
